# Inherited metabolic diseases mimicking hereditary spastic paraplegia: a cohort of ten Egyptian patients

**DOI:** 10.3389/fgene.2026.1963118

**Published:** 2026-10-22

**Authors:** Ola A. Khalifa, Tarek M. Youssef, Radwa G. Mahmoud

**Affiliations:** 1 Department of Medical Genetics, Faculty of Medicine, Ain Shams University, Cairo, Egypt; 2 Department of Internal Medicine, Faculty of Medicine, Ain Shams University, Cairo, Egypt

**Keywords:** hereditary spastic paraplegia (HSP), inborn errors of metabolism, neurometabolic disorders, rare diseases, targeted metabolic gene panels, whole exome sequencing

## Abstract

**Background:**

Inborn errors of metabolism (IEMs) are a rare and often underrecognized cause of spastic paraplegia in children and adults.Owing to their high energy demands and long axons, motor neurons and the corticospinal tracts are particularly vulnerable to metabolic defects. Consequently, several potentially treatable neurometabolic disorders can mimic both pure and complex hereditary spastic paraplegia (HSP). Although spasticity is a shared hallmark, these disorders differ in their genetic etiology, metabolic profiles, clinical manifestations, and treatment responses.

**Methods:**

We investigated 10 Egyptian patients with spastic paraplegia as the core clinical feature using an integrated approach combining clinical assessment, biochemical profiling, and molecular diagnostics.

**Results:**

We identified (3) pathogenic, (4) VUS, and (4) likely pathogenic variants in nine disease-causing genes (*SLC25A15, MTHFR, ARG1,OPA3, MOCS2, ETHE1, PANK2, C19orf12*, and *MTR*), including eight homozygous and two compound heterozygous genotypes, with seven novel variants. Patients’ ages ranged from 1.9 to 17 years, with a median symptom onset of 1.8 years and median diagnosis age of 9.25 years. Most of the patients (80%) were born to consanguineous parents. Gait disturbance/spasticity was the most common presenting feature (90%; 100% respectively). Abnormal Metabolic profile was reported in (5/10, 50%).

**Conclusion:**

Our findings highlight the value of integrated metabolic and genomic investigations in unexplained spastic paraparesis. Early molecular diagnosis enables precise disease identification, targeted therapy, and improved outcomes.

## 1 Introduction

Hereditary spastic paraplegia (HSP) represents a heterogeneous group of inherited neurodegenerative disorders characterized by progressive lower-limb spasticity and weakness resulting from corticospinal tract dysfunction (Saadi et al., 2025). Several inborn errors of metabolism (IEMs) can present with pure or complex HSP-like phenotypes and are potentially treatable conditions (Ebrahimi-Fakhari et al., 2022). Neurometabolic disorders that may mimic HSP include biotinidase deficiency, hyperornithinemia-hyperammonemia-homocitrullinemia syndrome, arginase deficiency, methylenetetrahydrofolate reductase deficiency, cerebrotendinous xanthomatosis, glucose transporter type 1 deficiency, metachromatic leukodystrophy, X-linked adrenoleukodystrophy, and dopa-responsive dystonia (Saadi et al., 2025). Differentiating these conditions can be challenging, as clinical features alone are often insufficient and require comprehensive biochemical and molecular evaluation (Teive et al., 2022). Early recognition is crucial, as targeted interventions such as dietary therapy, enzyme replacement, and cofactor supplementation may improve outcomes (Teive et al., 2022). This study emphasizes the role of metabolic assessment and genetic testing, including whole-exome sequencing, in patients with unexplained spastic paraparesis.

## 2 Patients and methods

This cross-sectional study included 10 Egyptian patients evaluated between November 2025 and April 2026 at the Metabolic Clinic, Medical Genetics Department, Ain Shams University. one of the largest national metabolic referral centers providing specialized healthcare services for patients with inborn errors of metabolism. Inclusion criteria were patients of all ages and both sexes presenting with progressive spastic paraplegia, with lower-limb spasticity as the predominant clinical feature. Exclusion criteria included a history suggestive of cervical spondylosis or radiculopathy; systemic diseases such as diabetes mellitus, hypertension, or cardiac, hepatic, or renal disease; previous trauma, surgery, or infection involving the brain or spinal cord; and other causes of spastic paraplegia, including spinal cord infarction, dopa-responsive dystonia, spinal cord malformations, or inflammatory disorders such as multiple sclerosis or vasculitis. Clinical assessment included detailed medical and family histories, anthropometric measurements, and neurological examination. Metabolic investigations included plasma total homocysteine, methylmalonic acid, ammonia, vitamin B12, and tandem mass spectrometry. Additional radiological, ophthalmological, and neurophysiological investigations were performed when clinically indicated. Molecular testing was performed using the CentoMetabolic® MOx panel, including NGS-based copy-number variant (CNV) analysis of 206 genes, for patients with suspected urea cycle disorders, while whole-exome sequencing (WES) was performed for patients without diagnostic clues or those with suspected neurometabolic disorders not covered by the panel, to establish or confirm the molecular diagnosis. WES targeted >98% of coding RefSeq exons based on the GRCh37/hg19 reference genome and included the mitochondrial genome. Variants were interpreted according to American College of Medical Genetics and Genomics (ACMG) criteria, with segregation analysis performed by Sanger sequencing.

• Statistical analysis: All data will be collected, tabulated and statistically analyzed by a commercially available software package program

### Case Presentation

Case 1: A 17-year-old boy, born to consanguineous Egyptian parents, presented with progressive gait disturbance and spasticity. Normal development was followed by protein aversion and recurrent vomiting at 15 months, then progressive falls, toe-walking, and shuffling gait from 10 years of age. Examination revealed microcephaly, lower-limb wasting, spasticity, hyperreflexia, and extensor plantar responses. Routine and specific metabolic investigations as well as neuroimaging were unremarkable. Whole-exome sequencing identified a homozygous likely pathogenic SLC25A15 variant, c.788delinsAAG; p.(Thr263Lysfs*8), confirming hyperornithinemia-hyperammonemia-homocitrullinemia (HHH) syndrome. Treatment with protein restriction, sodium benzoate, L‐arginine hydrochloride, and baclofen resulted in clinical stability and mild improvement of spasticity.
Case 2: A 12-year-old girl, born to consanguineous Egyptian parents, presented with progressive neurodevelopmental decline, gait disturbance, cognitive regression, and neuropsychiatric manifestations from 4 years of age. Examination showed lower-limb spasticity, hyperreflexia, and extensor plantar responses. Biochemical analysis revealed severe hyperhomocysteinemia (288 μmol/L), low methionine (3.5 μmol/L), and normal methylmalonic acid. Brain Magnetic Resonance Imaging (MRI) showed cerebral atrophy and demyelinating changes (Figure 1). Whole-exome sequencing identified a homozygous *MTHFR* variant of uncertain significance (VUS), c.560G > T; p.(Gly187Val), potentially associated with methylenetetrahydrofolate reductase (MTHFR) deficiency. Betaine therapy reduced tHcy to 114 μmol/L with biochemical improvement and clinical stabilization.
Case 3: A 7-year-old girl, born to consanguineous Egyptian parents, presented with progressive gait disturbance and developmental regression from 2 years and 2 months, including toe-walking, spasticity, and loss of skills. Examination revealed short stature, microcephaly, and lower-limb spasticity. Plasma arginine was markedly elevated (682 μmol/L) with normal ammonia. CentoMetabolic panel identified a homozygous likely pathogenic *ARG1* variant, c.947G > A; p.(Arg316GIn), confirming arginase-1 deficiency (ARG1-D). Treatment with rigorous dietary protein restriction reduced plasma arginine to 131 μmol/L with improvement in cognition, speech, and development; and baclofen improved spasticity.
Case 4: A 15-year-old boy, born to consanguineous Egyptian parents with an affected sibling (Case 3), presented with progressive spastic paraplegia. He developed frequent falls and toe walking at 2 years of age, with a history of marked protein aversion. His motor function progressively declined, resulting in loss of independent ambulation by 12 years, followed by partial improvement after orthopedic interventions. Examination revealed lower-limb spasticity, hypertonia, hyperreflexia, and bilateral extensor plantar responses. Metabolic evaluation showed markedly elevated plasma arginine level (590 μmol/L) with normal ammonia and lactate levels, confirming ARG1-D. Treatment with rigorous dietary protein restriction resulting in a significant reduction of plasma arginine levels during follow-up and baclofen improved spasticity.
Case 5: A 5-year-old boy, born to consanguineous Egyptian parents, presented with developmental delay and progressive neurological symptoms. Development was normal during the first year, followed by developmental plateau, abnormal movements, and gait instability from 18 months of age. Examination revealed lower-limb hypertonia, hyperreflexia, microcephaly, and bilateral optic atrophy with reduced visual acuity. Whole-exome sequencing identified a homozygous *OPA3* variant of uncertain significance (VUS) c.134C > G; p.(Ser45Cys), potentially associated with Costeff syndrome; however, urinary organic acid analysis showed no 3-methylglutaconic aciduria. The patient was lost to follow-up, limiting further clinical and biochemical evaluation.
Case 6: A 1.9-year-old girl, born to consanguineous Egyptian parents with a family history of a similarly affected sibling, developed fever, gastroenteritis, seizures, dystonia, and coma at 10 months, followed by developmental regression and recurrent metabolic decompensations. Whole-exome sequencing identified a homozygous pathogenic *MOCS2* variant c.3G > A; (p.Met1Ile), confirming autosomal recessive molybdenum cofactor deficiency type B. Examination showed severe growth failure, microcephaly, generalized hypertonia, and hyperreflexia with spastic parapressis, while brain MRI demonstrated diffuse cerebral atrophy. She was managed with a protein-restricted diet, levetiracetam, trihexyphenidyl, and biperiden. During follow-up, she showed improved seizure control and gradual developmental progress with acquisition of new milestones.
Case 7: A 4-year-old boy, born at term to non-consanguineous Egyptian parents after an uneventful pregnancy, had normal early development until 1 year of age, followed by developmental stagnation and progressive lower-limb stiffness beginning at 15 months. Examination showed failure to thrive, microcephaly, and spastic paraparesis with hypertonia, hyperreflexia, ankle clonus, and bilateral extensor plantar responses. Metabolic screening, plasma ammonia, lactate, and EEG were normal, while venous blood gas analysis revealed high anion gap metabolic acidosis. Brain Computed Tomography (CT) showed a deep right temporal cyst. Whole-exome sequencing identified two *ETHE1* variants c.406A > G; p.(Thr136Ala) likely pathogenic; c.121G > C; p.(Gly41Arg) variant of uncertain significance (VUS), confirming ethylmalonic encephalopathy (EE). Treatment with L-carnitine, riboflavin, coenzyme Q10, sodium bicarbonate, and metronidazole resulted in gradual developmental improvement and acquisition of new milestones during follow-up.
Case 8: An 8.5-year-old boy, born to consanguineous Egyptian parents, presented with progressive neurological and visual impairment. He developed toe walking and visual difficulties at 1.5 years, followed by night blindness, cone-rod dystrophy, progressive stiffness, dystonia, dysarthria, and dysphagia. Examination revealed upper-limb dystonia and lower-limb hypertonia with hyperreflexia. Brain MRI showed bilateral globus pallidus abnormalities with the characteristic “eye-of-the-tiger” sign (Figure 2). Ophthalmologic evaluation confirmed severe bilateral rod and cone dysfunction. Whole-exome sequencing identified a homozygous *PANK2* variant of uncertain significance (VUS) c.1594C > T; p.(Arg532Cys), potentially associated with pantothenate kinase-associated neurodegeneration (PKAN). The patient is receiving supportive management with physical and occupational therapy to preserve function and improve quality of life.
Case 9: A 13-year-old boy, born to consanguineous Egyptian parents, presented with progressive motor and neurodevelopmental impairment. He developed gait difficulty, frequent falls, toe walking, and speech disturbance at 5 years of age, followed by progressive deterioration. Examination revealed lower-limb hypertonia and hyperreflexia with preserved plantar responses. Brain MRI showed bilateral basal ganglia involvement consistent with neurodegeneration with brain iron accumulation (NBIA) (Figure 3). Ophthalmologic evaluation demonstrated bilateral optic disc pallor, macular and retinal nerve fiber layer thinning, and rod dystrophy on electroretinography. Whole-exome sequencing identified a homozygous pathogenic *C19orf12* variant c.204_214del; p.(Gly69ArgfsTer10), confirming autosomal recessive mitochondrial membrane protein-associated neurodegeneration (MPAN; NBIA type 4A). He is currently receiving baclofen, coenzyme Q10, and physiotherapy as supportive therapies, with a stable clinical course.
Case 10: A 15.5-year-old boy, born to non-consanguineous Egyptian parents, presented with delayed speech, poor school performance, progressive generalized hyperpigmentation, and lower-limb hypertonia and hyperreflexia. Laboratory evaluation revealed markedly elevated plasma homocysteine (150 μmol/L) with normal methylmalonic acid levels. Whole-exome sequencing identified compound heterozygous *MTR* variants c.3518C > T; p.(Pro1173Leu) pathogenic and c.412del; p.(Ile138Leufs*121) likely pathogenic, confirming methionine synthase deficiency (cblG-type homocystinuria). He was started on hydroxocobalamin, betaine anhydrous, L-carnitine, and folic acid therapy, with subsequent biochemical improvement, fading of hyperpigmentation, and improved academic performance following treatment adherence.

**FIGURE 1:**
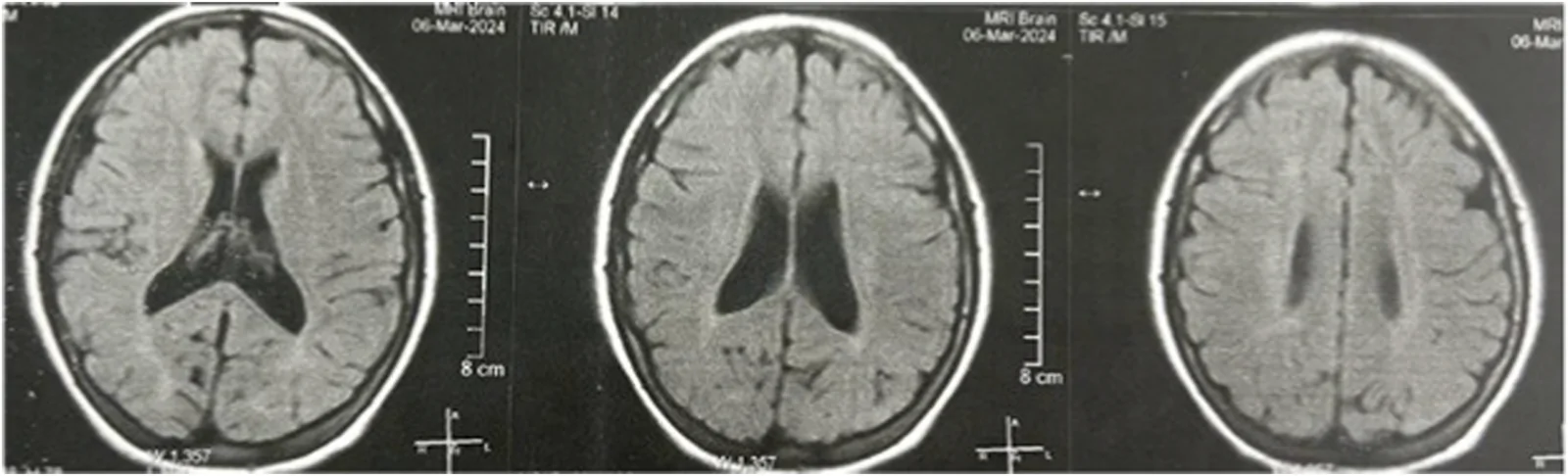
Brain MRI revealed generalized cerebral atrophic changes, bilateral periventricular white matter signal abnormalities suggestive of a demyelinating process, and mild dilatation of the ventricular system.

**FIGURE 2:**
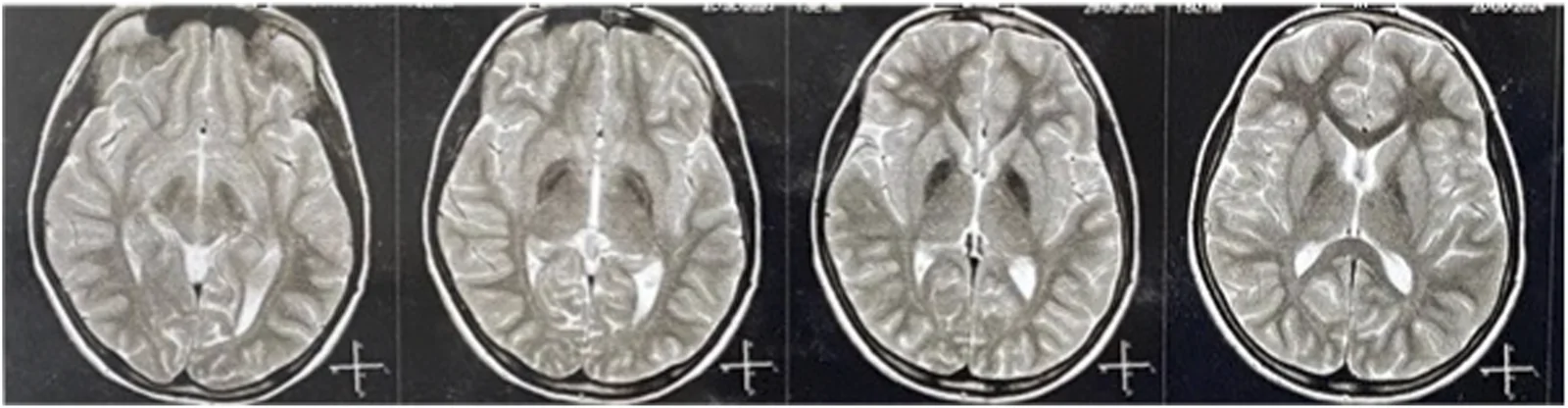
Brain MRI demonstrated bilateral basal ganglia signal abnormalities, with symmetrical central hyperintensity surrounded by hypointense signals in the globus pallidus, producing the characteristic “eye-of-the-tiger” sign.

**FIGURE 3:**
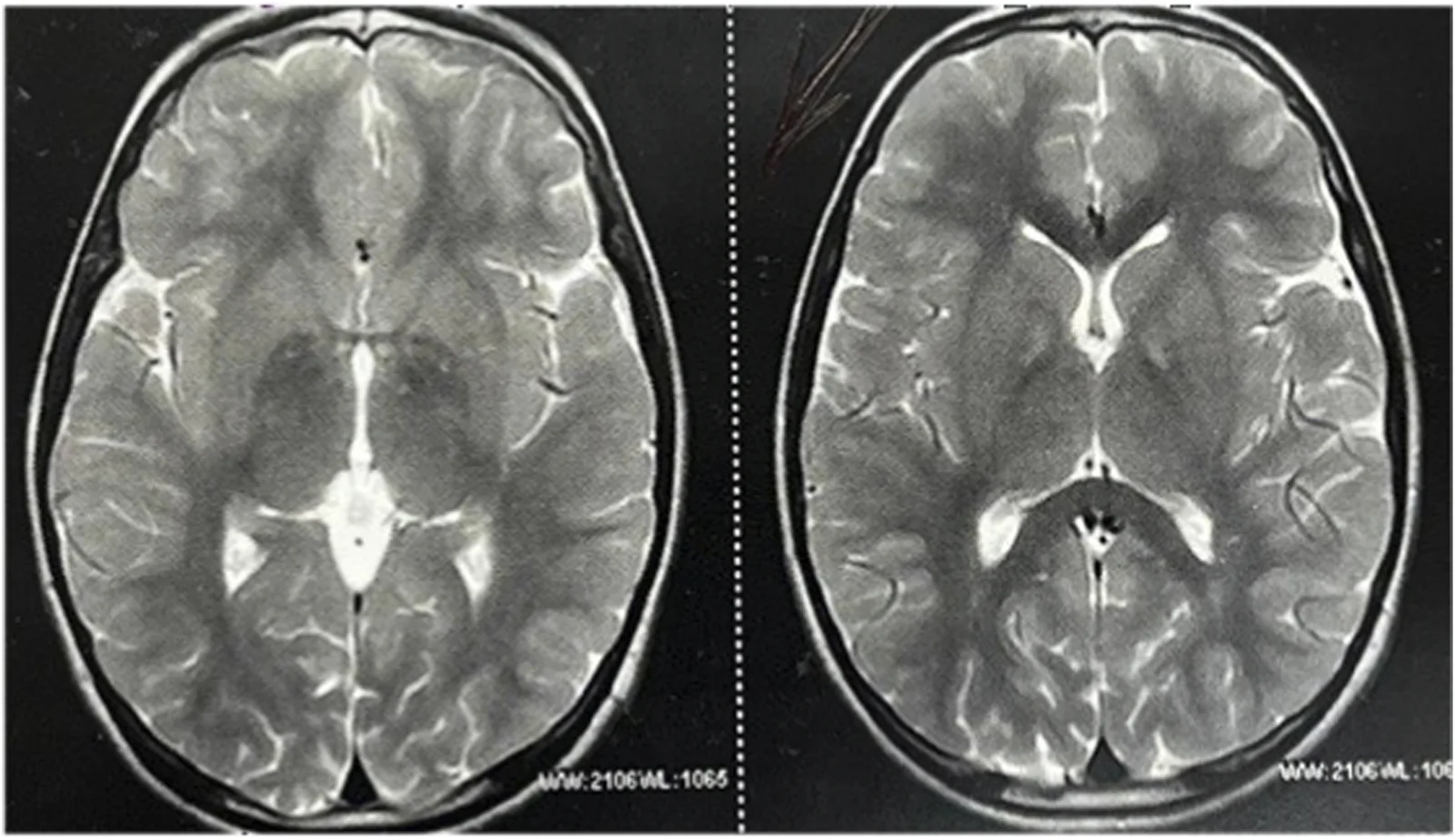
Brain MRI demonstrated bilateral basal ganglia involvement consistent with neurodegeneration with brain iron accumulation.

## 3 Results

This descriptive analytical study included 10 genetically confirmed Egyptian patients with metabolic disorders mimicking HSP, where spastic paraplegia was a core manifestation. The patients’ characteristics are illustrated in Table 1 as follows: patients’ ages ranged from 1.9 to 17 years, with a median symptom onset of 1.8 years and median diagnosis age of 9.25 years. The majority of patients (8/10,80%) were born to consanguineous parents (Figure 4). Some patients manifest more than one presenting feature and Table 1 shows that gait disturbance/spasticity was the most common presenting feature (9/10, 90%; 10/10,100% respectively), followed by psychomotor regression and cognitive impairment (4/10, 40% each). Additional manifestations were reported in some patients including protein aversion (3/10, 30%), speech problems (3/10, 30%), Psychiatric problems (1/10, 10%), ophthalmological/fundal abnormalities (3/10, 30%), skin manifestations (1/10, 10%). Abnormal Metabolic profile was positive in (5/10, 50%). Genetic diagnosis was achieved in all cases using either WES (8/10, 80%) or CentoMetabolic panel (2/10, 20%). Eleven variants were identified in nine disease-causing genes as illustrated in Table 2: (3) pathogenic, (4) likely pathogenic, and (4) VUS with seven novel variants. Homozygous variants were detected in eight patients, compound heterozygous variants in two patients.

**TABLE 1:** The characteristics of the studied patient**s**.

Case no.	Age (years)	Sex	Origin	Consanguinity	Age of starting symptoms (years)	Presenting picture	Age at diagnosis (years)	Biochemical findings	Treatment
1	17	M	E	+	1.3	Protein aversion, Usteady gait, progressive spasticity with pyramidal signs	16	EMS, ammonia, lactate, UOA and VBG were normal	Protein restriction, ammonia scavengers, antispastisity
2	12	F	E	+	4	Regression of previously acquired milestones of development, Psychiatric problems, spastic gait, cognitive. Decline	10	Plasma tHCy was elevated to 288 μmol/L, low methionine 3.5 μmol/L, normal MMA	Betaine anhydrous
3	7	F	E	+	2.2	Protein aversion, spastic gait, cognitive decline, regression of previously acquired milestones of development	6	Arginine 682 umol/L with normal ammonia	Protein restriction, antispasticity
4	15	M	E	+	2	Frequents falls, spastic gait, protein aversion, and cognitive decline	15	Arginine 590 umol/L with normal ammonia	Protein restriction, antispasticity
5	5	M	E	+	1.6	Abnormal movement, unsteadiness, spastic gait, decreased visual acuity	5	UOA was normal	supportive
6	1.9	F	E	+	0.8	convulsion, dystonia, spastic paraparesis and regression of previously acquired milestones of development	1.5	EMS, ammonia, lactate, and UOA were normal	protein restriction
7	4	M	E	-	1.3	Difficulty of walking with spastic paraparesis and stagnation of milestones of development	4	EMS, ammonia and lactate were normal, metabolic acidosis with high anion gab	L carnitine, co q10, .,Nahco3, metronidazole
8	8.5	M	E	+	1.5	Vision problems, progressive spasticity with pyramidal signs dysarthria, dystonia, and dysphagia	8.5	EMS, ammonia, lactate were normal	Supportive
9	13	M	E	+	5	difficulty in walking, frequent falling, tip toe walking, spastic gait, dysarthria regression of previously acquired milestones, rod dystrophy and optic nerve pallor	13	EMS, ammonia, lactate were normal	Supportive
10	15.5	M	E	-	5	Delayed speech,poor school achievement cognitive decline spastic gait, hyperpigmentation	15	Plasma tHCy was elevated, normal MMA	B12, betaine, L-carnitine, and folic acid therapy

No: Number; M: Male, F: Female, E: Egyptian,+: positive; EMS: Extended metabolic screening,VBG: Venous blood gas; UOA: Urine organic acid; tHCy: Total homocysteine; MMA: Methylmalonic acid.

**FIGURE 4:**
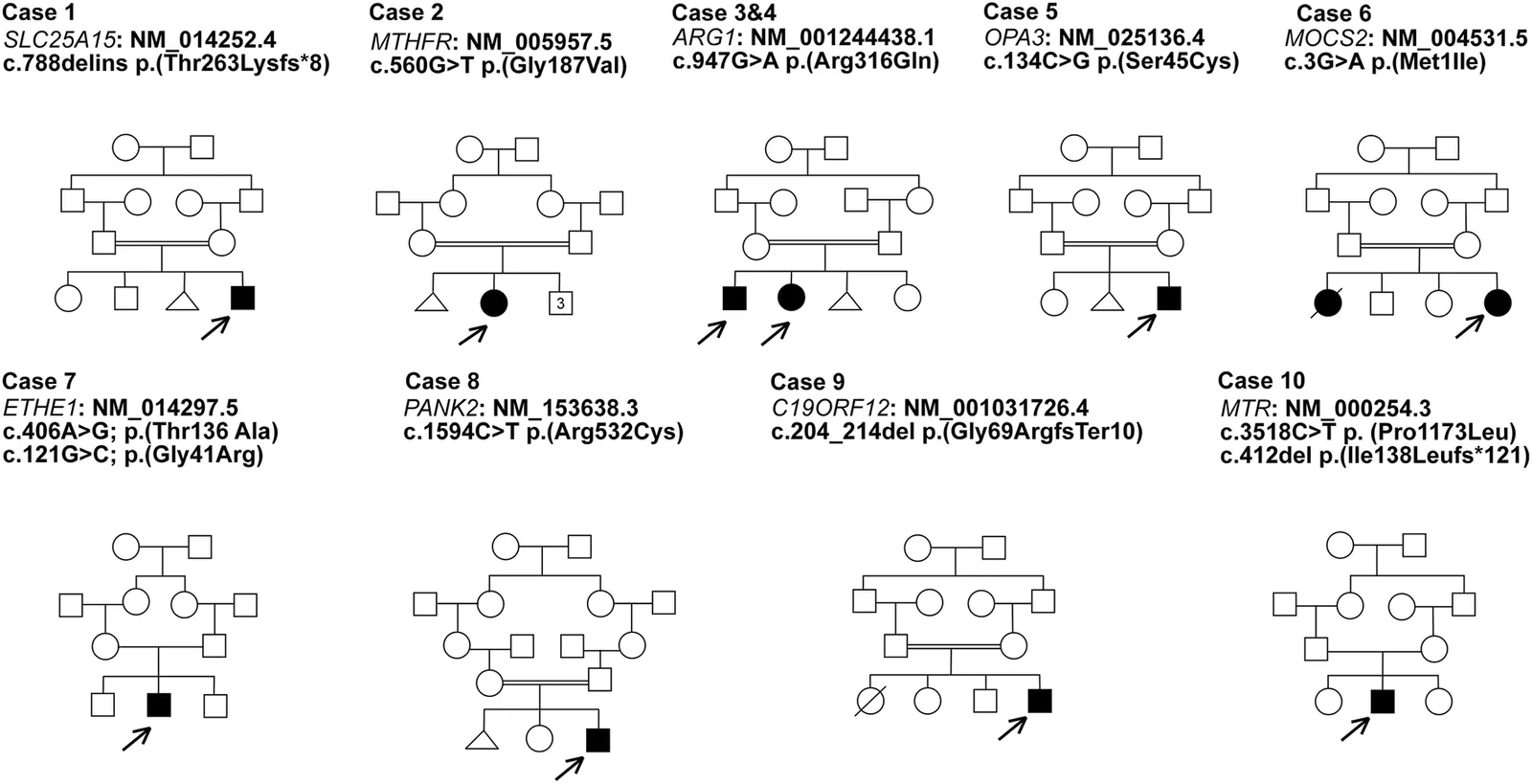
Pedigrees of nine families with neurometabolic disorders. Pedigrees illustrate affected individuals (filled symbols), and unaffected relatives (open symbols). The proband in each family is indicated by an arrow. Identified variants are annotated next to the corresponding pedigree.

**TABLE 2:** Molecular characterization of the studied patients.

Case no.	Gene	Transcription position	Variant	Zygosity	ACMG classification	References
1	SLC25A15	NM_014252.4	c.788delinsAAG; p. (Thr263Lysfs*8)	Homozygous	likely pathogenic	This Study
2	MTHFR	NM_005957.5	c.560G > T; p.(Gly187Val)	Homozygous	VUS	This Study
3	ARG1	NM_001244438.1	c.947G > A; p. (Arg316GIn)	Homozygous	likely pathogenic	This Study
4	ARG1	NM_001244438.1	c.947G > A; p. (Arg316GIn)	Homozygous	likely pathogenic	This Study
5	OPA3	NM_025136.4	c.134C > G; p.(Ser45Cys)	Homozygous	VUS	This Study
6	MOCS2	NM_004531.5	c.3G > A; (p.Met1Ile)	Homozygous	Pathogenic	Reiss et al. (1999)
7	ETHE1	NM_014297.5	c.406A > G; p.(Thr136Ala)	Heterozygous	Likely Pathogenic	Tiranti et al. (2006)
		NM_014297.5	c.121G > C; p.(Gly41Arg)	Heterozygous	VUS	This Study
8	PANK2	NM_153638.3	c.1594C > T; p.(Arg532Cys)	Homozygous	VUS	This Study
9	C19orf12	NM_001031726.4	c.204_214del (p.Gly69ArgfsTer10	Homozygous	Pathogenic	Hartig et al. (2011)
10	MTR	NM_000254.3	c.3518C > T; p.(Pro1173Leu)	Heterozygous	Pathogenic	Gulati et al. (1996)
		NM_000254.3	c.412del (p.Ile138Leufs*121)	Heterozygous	Likely pathogenic	This Study

VUS: Variant of uncertain significance.

## 4 Discussion

Several inborn errors of metabolism (IEMs) can mimic pure or complex HSP phenotypes and represent potentially treatable conditions (Ebrahimi-Fakhari et al., 2022). Common neurometabolic mimics include cerebrotendinous xanthomatosis, arginase-1 deficiency, methylenetetrahydrofolate reductase deficiency, metachromatic leukodystrophy, and X-linked adrenoleukodystrophy (Saadi et al., 2025). Due to overlapping clinical manifestations, comprehensive biochemical and molecular evaluation is essential for accurate diagnosis (Rohatgi et al., 2023; Teive et al., 2022).

A substantial diagnostic delay was observed in our cohort, with molecular confirmation obtained only after years of neurological progression, partly due to the high cost and limited availability of genomic testing in local hospitals. Similar barriers to molecular diagnosis have been reported in HSP, where the cost and accessibility of genetic testing have historically limited diagnostic evaluation, particularly before the wider availability of next-generation sequencing (Hensiek et al., 2015; Blackstone, 2022). Furthermore, the clinical and genetic heterogeneity of HSP and its overlap with treatable inherited metabolic disorders can further contribute to delayed recognition (Schiavoni et al., 2020; Cao et al., 2024; Assaedi et al., 2025; Davarzani et al., 2026). Expanding access to affordable targeted molecular panels or reduced-cost genetic testing programs may help overcome these financial barriers and facilitate earlier diagnosis, particularly in resource-limited settings. These findings emphasize the importance of early metabolic evaluation and timely genomic testing in patients with atypical or unexplained spastic paraplegia to identify potentially treatable conditions.

The high rate of parental consanguinity in our cohort likely contributed to the predominance of autosomal-recessive neurometabolic disorders, a pattern frequently observed in Egyptian and other Middle Eastern populations (Abdelkhalek et al., 2024). These findings, together with regional data on the genetic spectrum of HSP (Salari et al., 2025), are consistent with our cohort and support prioritizing early metabolic and genetic evaluation in populations with high rates of consanguinity.

Urea cycle disorders represented an important subgroup in our cohort. Patients with HHH syndrome and ARG1-D presented mainly with progressive spastic paraplegia without significant hyperammonemic crises, consistent with previous reports identifying these disorders as treatable HSP mimics (Olivieri et al., 2019; Ebrahimi-Fakhari et al., 2022).

The patient with HHH syndrome carried a novel homozygous *SLC25A15* frameshift variant, c.788delinsAAG (p.Thr263Lysfs*8). Early protein aversion and vomiting were noted; however, normal routine and specific metabolic investigations, together with the absence of overt metabolic crises, contributed to diagnostic delay. Notably, the classical biochemical hallmark of HHH syndrome was absent in our patient. This finding is consistent with previous reports describing incomplete or atypical biochemical phenotypes in some HHH syndrome patients (Martinelli et al., 2015; Olivieri et al., 2019). In agreement with previous studies, our results emphasize that HHH syndrome should be considered in the differential diagnosis of progressive spastic paraplegia, even when specific metabolic investigations are unremarkable, as early recognition and treatment may help limit neurological progression (Martinelli et al., 2015; Olivieri et al., 2019).

Similarly, two siblings with ARG1-D carried a novel homozygous ARG1 variant, c.947G > A (p.Arg316Gln), presenting with progressive spastic paraplegia and cognitive decline. Their presentation initially mimicked complicated hereditary spastic paraplegia (HSP). The red flags of ARG1-D such as pronounced worsening of spasticity, cognitive and motor decline after the first year of life and avoidance of high protein food may provide valuable clues for maintaining a high index of suspicion and facilitating early diagnosis (Freua et al., 2022). In our cohort, recognition of these features in (case 3) contributed to the early clinical suspicion of ARG1-D. Accurate diagnosis of these disorders requires integration of clinical assessment, family history, and appropriate biochemical and genetic testing, as illustrated by our cohort, where the diagnosis in (case 4) was established following the evaluation of his sister (case 3). Therefore, experts recommended routine assessment and inclusion of ARG1 gene in molecular panels for differential diagnosis of HSP (Freua et al., 2022; McNutt et al., 2022; Burlina et al., 2025; Castellanos-Pinedo et al., 2026).

MTHFR deficiency is a rare remethylation disorder with variable presentations, including late-onset HSP-like phenotypes. The patient with a novel homozygous *MTHFR* variant, c.560G > T (p.Gly187Val), presented with spastic paraplegia, cognitive decline, behavioral abnormalities, and marked hyperhomocysteinemia. Elevated homocysteine, low methionine, and normal methylmalonic acid levels supported a remethylation defect, further confirmed by neuroimaging findings (Diekman et al., 2014; Zeltner et al., 2014; Huemer et al., 2017). Betaine therapy resulted in biochemical improvement and clinical stabilization, consistent with previous reports demonstrating treatment benefit (Martinelli et al., 2015; Olivieri et al., 2026). Therefore, HSP like presentations with cognitive abnormalities should lead to assessment of homocysteine and methionine as MTHFR deficiency is potentially treatable.

The patient with Costeff syndrome carried a novel homozygous *OPA3* variant, c.134C > G (p.Ser45Cys), presenting with optic atrophy, developmental regression, and spasticity despite normal urinary organic acid analysis. Costeff syndrome is an OPA3-related mitochondrial disorder characterized by childhood-onset optic atrophy, reduced visual acuity, nystagmus, and later development of spastic paraparesis, ataxia, extrapyramidal manifestations, and variable cognitive impairment (Jones et al., 2021; Temizyürek et al., 2023). Although elevated urinary 3-methylglutaconic and 3-methylglutaric acids are characteristic, normal urinary organic acid analysis has been reported in genetically confirmed cases and does not exclude the diagnosis, as observed in our patient (Jones et al., 2021). This case highlights the importance of recognizing the combination of optic atrophy, cognitive abnormalities, regression, and spasticity as diagnostic clues for Costeff syndrome despite normal metabolic screening.

The patient with MoCD-B carried the homozygous *MOCS2* variant c.3G > A (p.Met1Ile), previously reported in MoCD-B by Reiss et al. (1999), and Crenshaw et al. (2026). The recurrence of this variant and the compatible neurological phenotype in our patient further supports its pathogenicity. Although MoCD-B typically presents with neonatal encephalopathy and seizures, later-onset phenotypes with developmental regression and progressive spasticity or spastic paraplegia have also been described (Schwahn et al., 2024), consistent with our patient. Early recognition is important because timely treatment may limit irreversible neurological injury, whereas cysteine-restricted therapy is unlikely to reverse established damage (Schwahn et al., 2024).

The patient with EE carried compound heterozygous *ETHE1* variants, including c.406A > G; p.(Thr136Ala) and c.121G > C; p.(Gly41Arg), presenting with progressive neuroregression with spasticity. Despite metabolic screening, as reported in some previous cases, the clinical phenotype prompted molecular testing, confirming the diagnosis and highlighting that normal biochemical markers do not exclude EE (Govindaraj et al., 2020). Therefore, EE should be considered in children presenting with neuroregression and progressive spasticity, even in the absence of characteristic biochemical abnormalities, as early molecular diagnosis is crucial for timely and appropriate management.

NBIA disorders included PKAN and MPAN, both presenting with progressive spasticity, dystonia, retinal involvement, and characteristic MRI findings. The PKAN patient carried a novel homozygous *PANK2* variant, c.1594C > T (p.Arg532Cys), while the MPAN patient carried the pathogenic *C19orf12* frameshift variant, c.204_214del (p.Gly69ArgfsTer10). These cases highlight the importance of considering targeting genetic testing for NBIA disorders in children with spasticity, dystonia, and visual abnormalities and characteristic brain MRI findings (Gregory et al., 2019; Tao et al., 2023; Gnutti et al., 2025).

The patient with methionine synthase deficiency (cblG-type homocystinuria) carried compound heterozygous *MTR* variants, including c.3518C > T (p.Pro1173Leu) and c.412del (p.Ile138LeufsTer121) presenting with cognitive decline, epilepsy, pyramidal signs, and severe hyperhomocysteinemia without hematological abnormalities. This highlights the variable presentation of cblG and the importance of assessing homocysteine levels in unexplained neurological disorders (Ferreira et al., 2026).

Several identified disorders were treatable, with targeted interventions leading to biochemical improvement and clinical stabilization. These findings underscore the value of integrating metabolic and genomic screening into unexplained spastic paraplegia, particularly in consanguineous populations. In the absence of universal newborn screening for treatable IEMs in Egypt, early recognition of neurometabolic mimics of HSP is essential to enable timely, potentially disease-modifying treatment.

## 5 Conclusion

IEMs represent a key group of treatable HSP mimics and should be considered in patients with progressive spasticity and unexplained neurological features Early metabolic and molecular evaluation is crucial for timely diagnosis, therapeutic intervention, and improved neurological outcomes. A limitation of this study is the identification of VUS; although all were supported by concordant clinical, biochemical, and/or neuroimaging findings, further functional and segregation studies are needed to establish pathogenicity. Nevertheless, these findings may contribute to future variant reclassification.

## Data Availability

The original contributions presented in the study are included in the article/Supplementary Material, further inquiries can be directed to the corresponding author.
