# Supplementary material for: Inherited metabolic diseases mimicking hereditary spastic paraplegia: a cohort of ten Egyptian patients
Source: Front Genet. 2026 Oct 22;17:1963118. doi: 10.3389/fgene.2026.1963118 (PMC13735532; doi:10.3389/fgene.2026.1963118)
Supplement: Supplementary file 1 [file Table1.docx]

**Table 1:** **The characteristics of the studied patients**

| **Case No.** | **Age (years)** | **Sex** | **Origin** | **Consanguinity** | **Age of starting symptoms (years)** | **Presenting picture** | **Age at diagnosis (years)** | **Biochemical findings** | **Treatment** |
| --- | --- | --- | --- | --- | --- | --- | --- | --- | --- |
| 1 | 17 | M | E | + | 1.3 | Protein aversion, Usteady gait, Progressive spasticity with pyramidal signs | 16 | EMS, ammonia, lactate, UOA and VBG were normal | Protein restriction, ammonia scavengers, antispastisity |
| 2 | 12 | F | E | + | 4 | Regression of previously acquired milestones of development, Psychiatric problems, spastic gait, cognitive. Decline, | 10 | Plasma tHCy was elevated to 288 μmol/L, low methionine 3.5 μmol/L, normal MMA | Betaine anhydrous |
| 3 | 7 | F | E | + | 2.2 | Protein aversion, spastic gait, cognitive decline, regression of previously acquired milestones of development | 6 | Arginine 682 umol/l with normal Ammonia. | Protein restriction, antispasticity |
| 4 | 15 | M | E | + | 2 | Frequents falls, Spastic gait , protein aversion, and cognitive decline | 15 | Arginine 590 umol/l with normal Ammonia | Protein restriction, antispasticity |
| 5 | 5 | M | E | + | 1.6 | Abnormal movement, unsteadiness, spastic gait, decreased visual acuity | 5 | UOA was normal | supportive |
| 6 | 1.9 | F | E | + | 0.8 | convulsion, dystonia, spastic paraparesis and regression of previously acquired milestones of development | 1.5 | EMS, ammonia, lactate , & UOA were normal | protein restriction |
| 7 | 4 | M | E | - | 1.3 | Difficulty of walking with spastic paraparesis and stagnation of milestones of development | 4 | EMS, ammonia and lactate were normal, metabolic acidosis with high anion gab. | L carnitine, co q10, .,Nahco3 , metronidazole |
| 8 | 8.5 | M | E | + | 1.5 | Vision problems, Progressive spasticity with pyramidal signs dysarthria, dystonia, & dysphagia | 8.5 | EMS, ammonia, lactate were normal; | Supportive |
| 9 | 13 | M | E | + | 5 | difficulty in walking , frequent falling, tip toe walking, spastic gait, dysarthria regression of previously acquired milestones, rod dystrophy & optic nerve pallor | 13 | EMS, ammonia, lactate were normal | Supportive |
| 10 | 15.5 | M | E | - | 5 | Delayed speech ,poor school achievement cognitive decline spastic gait, hyperpigmentation | 15 | Plasma tHCy was elevated, normal MMA | B12, betaine, L-carnitine, and folic acid therapy |

No : number; M: male, F : female, E: Egyptian,+ : positive; EMS : extended metabolic screening,VBG: Venous blood gas; UOA: Urine organic acid; tHCy: Total homocysteine; MMA: Methylmalonic acid
