# Supplementary material for: Inherited metabolic diseases mimicking hereditary spastic paraplegia: a cohort of ten Egyptian patients
Source: Front Genet. 2026 Oct 22;17:1963118. doi: 10.3389/fgene.2026.1963118 (PMC13735532; doi:10.3389/fgene.2026.1963118)
Supplement: Supplementary file 2 [file Table2.docx]

**Table 2: Molecular characterization of the studied patients**

| **Case No.** | **Gene** | **Transcription position** | **Variant** | **Zygosity** | **ACMG Classification** | **References** |
| --- | --- | --- | --- | --- | --- | --- |
| 1 | SLC25A15 | NM_014252.4 | c.788delinsAAG; p. (Thr263Lysfs*8) | Homozygous | likely pathogenic | This Study |
| 2 | MTHFR | NM_005957.5 | c.560G>T; p.(Gly187Val) | Homozygous | VUS | This Study |
| 3 | ARG1 | NM_001244438.1 | c.947G>A; p. (Arg316GIn) | Homozygous | likely pathogenic | This Study |
| 4 | ARG1 | NM_001244438.1 | c.947G>A; p. (Arg316GIn) | Homozygous | likely pathogenic | This Study |
| 5 | OPA3 | NM_025136.4 | c.134C>G; p.(Ser45Cys) | Homozygous | VUS | This Study |
| 6 | MOCS2 | NM_004531.5 | c.3G>A; (p.Met1Ile) | Homozygous | Pathogenic | (Reiss et al.,1999) |
| 7 | ETHE1 | NM_014297.5 | c.406A>G; p.(Thr136Ala) | Heterozygous | Likely Pathogenic | (Tiranti et al., 2006) |
|  |  | NM_014297.5 | c.121G>C; p.(Gly41Arg) | Heterozygous | VUS | This Study |
| 8 | PANK2 | NM_153638.3 | c.1594C>T; p.(Arg532Cys) | Homozygous | VUS | This Study |
| 9 | C19orf12 | NM_001031726.4 | c.204_214del (p.Gly69ArgfsTer10 | Homozygous | Pathogenic | (Hartig et al.,2011) |
| 10 | MTR | NM_000254.3 | c.3518C>T; p.(Pro1173Leu) | Heterozygous | Pathogenic | ( Gulati et al.,1996) |
|  |  | NM_000254.3 | c.412del (p.Ile138Leufs*121) | Heterozygous | Likely pathogenic | This Study |

VUS: Variant of uncertain significance
